# Flow development and leading edge vorticity in bristled insect wings

**DOI:** 10.1007/s00359-023-01617-x

**Published:** 2023-02-22

**Authors:** Felicity O’Callaghan, Fritz-Olaf Lehmann

**Affiliations:** Department of Animal Physiology, Institute of Biosciences, University of Rostock, Albert-Einstein-Str. 3, 18059 Rostock, Germany

**Keywords:** Insect flight, Bristled wings, Unsteady aerodynamics, Leading edge vortex, Particle image velocimetry

## Abstract

Small flying insects such as the tiny thrip *Gynaikothrips ficorum* have wings with bristles attached to a solid shaft instead of solid membranes. Air passing through the bristle fringe, however, makes bristled insect wings less effective for aerodynamic force production. In this study, we quantified the ability of bristled wings to generate a leading edge vortex (LEV) for lift support during wing flapping, scored its circulation during wing translation, and investigated its behaviour at the stroke reversals. The data were measured in robotic model wings flapping with a generic kinematic pattern at Reynolds number of ~ 3.4, while applying two-dimensional particle image velocimetry. We found that aerodynamic performance due to LEV circulation linearly decreases with increasing bristle spacing. The wings of *Gynaikothrips ficorum* might thus produce approximately 9% less aerodynamic force for flight than a solid membranous wing. At the stroke reversals, leading and trailing edge vortices dissipate quickly within no more than ~ 2% of the stroke cycle duration. This elevated dissipation makes vortex shedding obsolete during the reversals and allows a quick build-up of counter-vorticity when the wing reverses flapping direction. In sum, our findings highlight the flow conditions associated with bristled wing design in insects and are thus significant for assessing biological fitness and dispersal of insects flying in a viscosity-dominated fluid regime.

## Introduction

Flying insects have body sizes ranging from the centimetre to the micrometer scale. The ability to fly is remarkable in the smallest insects, because viscous air friction on the body and flapping wings distinctly attenuates aerial locomotion. Body miniaturization also leads to physiological constraints on the sensorimotor system owing to a reduced number of receptors and neurons needed for sensory processing (e.g. Polilov 2012; Huber and Noyes 2013). Small eyes, for example, have a reduced image resolution making vision-guided flight control more difficult (Land 1997). In general, a large number of species is evolved with similar morphology but different physiology due to the difference in body size (scaling effect). The genetic modifications necessary to enlarge or shrink the body of an insect species are often simple and typically only result from changes in expression levels of the steroid molting hormone ecdysone (Nijhout et al. 2006). Small body size is typically initiated by an early, single pulse of ecdysone causing larvae to stop feeding and initiate metamorphosis.

The consequences of size changes for locomotor behaviour in insects may be dramatic, in particular for flight, owing to the change in Reynolds number. As the body becomes progressively smaller, the relative effect of air viscosity on friction increases, resulting in proportionally higher drag and lower lift production (O’Callaghan et al. 2022). This leads to elevated energetic costs for flight, which is the most costly form of animal locomotion. Metabolic activity of insect flight muscles is thus the highest among the animal kingdom (Ellington 1991; Marden 2000; Lehmann 2001). In large insect species such as locusts, flies, moths, and bees, inertial costs for wing flapping are significant compared to aerodynamic costs because of the large wing mass and added mass acceleration at the beginning of each half stroke. Nevertheless, large insects flying at intermediate or elevated Reynolds numbers benefit two-fold compared to their smallest relatives: the former fly at high lift-to-drag ratios and may minimize inertial costs by means of elastic energy storage (Dickinson 1995). Since the ability of an insect to fly long distances depends on the efficient use of energy reserves, miniature insects should be severely limited in their locomotor capacity, for example to bridge the distance between two host plants. Thus, in small insects, long dispersal distances between geographical regions are often the result of passive drifting with wind currents (Freeman 1991) or hitch-hiking on larger insects (Fatouros and Huigens 2012; Houck and O’Connor 1991).

**Fig. 1:**
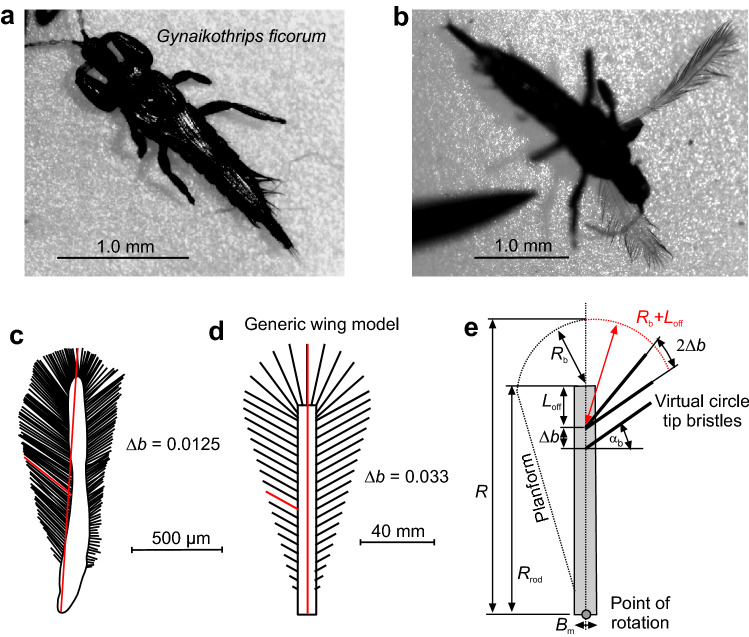
Bristled wings in the thrip *Gynaikothrips ficorum* and wing model reconstruction. **a, b** Photographs of the natural archetype showing body shape and dense bristles attached to a pronounced inner wing shaft. Images by courtesy of Gal Ribak, Tel Aviv University. **c** Drawing of the thrip wing. **d** Model wing showing number and orientation of the bristles. Red lines indicate the wing’s longitudinal axis and bristle length, respectively. **e** Construction of model wings used in this study with *R* = 125 mm, wing length; *R*_rod_ = 100 mm, length of membranous core; *B*_m_ = 8.6 mm, width of wing base; α_b_ = 29°, bristle angle; and ∆*b*, bristle spacing. Model parameters have been published previously (see Table 2 in O’Callaghan et al. 2022)

Small flying insects often exhibit special morphological adaptations such as ptiloptery, which refers to wings with long bristles attached to a narrow central shaft or membrane (Fig. 1; Horridge 1956; Polilov 2015; Rensch 1948; Rohdendorf 1949; Sane 2016). The advantage of ptiloptery is that it lowers wing mass and thus potentially lowers inertial power requirements for wing flapping (Weihs and Barta 2007), although this assumption has never been experimentally proven in many insect species. The disadvantage of bristled wings is that they produce less aerodynamic force compared to fully membranous wings, because air may pass through the wing surface (Kolomenskiy et al. 2020; Kasoju et al. 2018). Theoretical models predict that the performance of a bristled wing decreases with increasing bristle spacing (Kolomenskiy et al. 2020; Kasoju et al. 2018). Nevertheless, bristled wings could act in a similar way to a solid membrane, if the spacing of bristles is appropriate (Cheer and Koehl 1987). Compared to solid wing aerodynamics, there is relatively little known about aerodynamic performance of bristled wings and the flow structures associated with these morphological modifications. A previously published study on model wings with different bristle spacing has shown that lift coefficients decrease with increasing bristle spacing in a non-linear fashion (O’Callaghan et al. 2022). At a relative bristle spacing of 0.08 wing length (solid wing, 1.0), a bristled wing loses the ability to produce positive lift while drag is pronounced, yielding drag coefficients around 3.0 due to viscous friction. By contrast, the leakiness of bristled wings is thought to be beneficial in gusty exterior flows by dampening aerodynamic force peaks compared to a solid wing surface (Lee and Kim 2021). Leakiness may also help to reduce high drag on wings during clap-and-fling kinematics at the dorsal stroke reversal when left and right wings are pulled apart at the beginning of the downstroke (Santhanakrishnan et al. 2014).

It remains somewhat puzzling that bristles may also augment the lift-to-drag ratio and thus flight performance in wings containing 15–30% solid membrane and 70–85% bristles (Ford et al. 2019). Some miniaturized bristle-winged insects such as the tiny dwarf beetle *Paratuposa placentis*, for example, fly at speeds and accelerations of insects three times as large (Farisenkov et al. 2021). The beetle's body length is only 395 μm and its body mass 2.43 µg. The beetle flaps its bristled wings in a figure-of-eight loop. The flapping cycle is functionally decomposed into two power half strokes producing a large upward force and two negative lift-producing recovery half strokes. Inertial power requirements are small owing to the extremely small mass of the lift-producing hind wings (~ 0.0235 µg). Compared to membranous wings, the bristled structure of the beetle wings nearly maintains the same aerodynamic efficiency (Farisenkov et al. 2021). Reynolds number in miniature insects is typically below unity for flows around individual bristles, while flow that encompasses the entire wing is typically scattered around values below ~ 10 (Davidi and Weihs 2012; Kolomenskiy et al. 2020). Analytical studies show that flow between adjacent bristles depends on bristles’ diameter and the space between them including flow velocity (Cheer and Koehl 1987). Bristle density should thus be a factor that determines bound circulation and leading edge vorticity (leading edge vortex, LEV) in root-flapping insect wings. Numerous previously published studies on solid membranous wings in insects have shown how LEVs enhance total circulation and thus lift production during flapping at Reynolds numbers ranging from ~ 100 to ~ 10.000 (e.g. Sane 2003; Shyy et al. 2008, 2016).

To extend our knowledge on flow development in tiny insect wings, this study quantifies the ability of bristled model wings to produce a leading edge vortex with varying bristle spacing and while flapping at low Reynolds number of ~ 3.4. The tested wings are modelled on the wings of the 1.5 mm thrip *Gynaikothrips ficorum* (Fig. 1a, b). We scored the time evolution of the leading edge vortex during wing flapping, studied vortex dissipation and investigated how the magnitude of LEV vorticity changes the ability of a bristled wing to produce vertical force for weight support. The materials and methods used in this study are similar to a previously published study on bristle wing aerodynamics, in which we used bristled robotic wings to estimate lift and drag coefficients, including Froude efficiency of propulsion (O’Callaghan et al. 2022).

## Materials and methods

### Wing design and kinematics

**Fig. 2:**
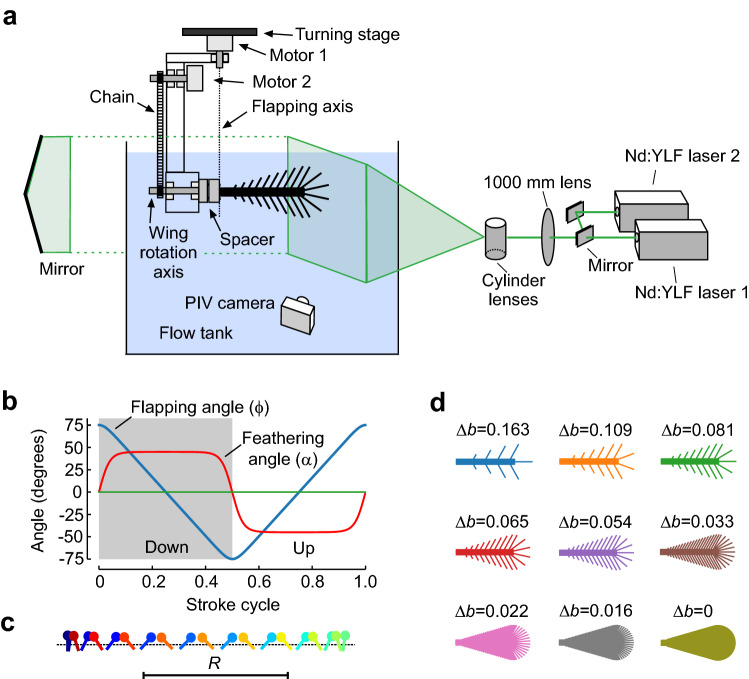
Experimental setup, wing kinematics and tested wings. **a** Wing models are flapped by two servo motors for horizontal flapping and wing rotation. The wings were immersed in a 60 × 40 × 36 cm^3^ tank filled with glycerine and flapped ~ 6 cm below the fluid surface to avoid wave generation. A turning stage allows manual rotation of the flapper and orientation of the wing towards the camera for particle image velocimetry (PIV). The tank is seeded with small air bubbles. **b** A generic lift-based kinematic with the wing's feathering angle (red), horizontal flapping angle (blue), and vertical heaving angle (green). **c** Lollipop diagram of a wing chord in *xy*-space with markers indicating the leading edge of the wing. The different times of the stroke cycle are colour-coded. **d** Cartoons of the tested solid (membranous) and bristled wings

The simplified wings used in this study are identical to the wings previously used in numerical (Engels et al. 2021) and experimental approaches (O’Callaghan et al. 2022). Wing design and kinematic pattern are explained in great detail in the latter manuscripts, and we thus refer to the materials and methods section of both studies for further information. Here, we only present a short description of the experimental approach. The snow cone-like model wings were printed from polyvinylalcohol plastic (PVA) using a high-resolution Ultimaker3 3D printer (Ultimaker, Utrecht, the Netherlands) with Cura 4.8 software. The wings had a narrow root, different number of bristles with varying spacing (Δ*b*), attachment sites and area coverage (Fig. 1d, e). We defined bristle spacing Δ*b* as the mean distance between two bristle tips of natural wings but excluded 20% of the smallest and largest bristles. We tested wings with a ∆*b* (number of bristles) of 0.163 (9), 0.109 (14), 0.081 (19), 0.065 (24), 0.054 (29), 0.033 (49), 0.022 (74), 0.016 (99) and 0 (fully membranous, Fig. 2d). These values are equal to a relative covered area of approximately 0.21, 0.23, 0.24, 0.26, 0.28, 0.34, 0.42, 0.51 and 1.0, respectively (O’Callaghan et al. 2022). The wing shaft covers ~ 20% area of the fully membranous wing. Wing thickness was ~ 2.0 mm at the base and ~ 1.3 mm at the end of the membranous core. Bristle diameter was ~ 1.1 mm or ~ 8.8 $\times$ 10^–3^ wing length and bristle orientation relative to the wing's longitudinal axis was ~ 29° (Fig. 1e). The wing with Δ*b* = 0.016 was similar to the natural archetype of *Gynaikothrips ficorum* that has 173 bristles, a bristle angle of 54.9°, a bristle spacing of 0.0125*R*, and 0.101*R* bristle length (wing length, *R*; Fig. 1c, d). In the thrip, wingbeat frequency is 196 Hz and Reynolds number *Re* = 24 (see below for *Re* of model wings). The 3d-printed bristles were made rather rigid, because real wing bristles are remarkably stiff (Jiang et al. 2022) and a theoretical model predicts only negligible bristle bending during wing flapping motion (Engels et al. 2021). The wings were flat, because the contribution of wing corrugation to net aerodynamic forces is minimal in insect wings (Engels et al. 2020).

For testing, the wings were immersed in a flow tank (~ 60 cm width, ~ 36 cm height, and ~ 40 cm depth) filled with glycerine (density, ~ 1260 kg m^−3^) and flapped by two small commercial servo motors (Graupner DS-series, Graupner, Germany) and a timing belt (Fig. 2a). The motors were position-controlled by an ATmega16L micro-controller on a STK500 board (Microchip AVR, Chandler, Arizona, USA). Motors and wing mechanics were mounted to a laser-calibrated turning stage that let us position the wing’s reference position at arbitrary angles towards the camera. This allowed us to map the wake throughout the entire stroke cycle. Each wing flapping sequence consisted of 10 consecutive stroke cycles with ~ 15 s resting periods in between, allowing the fluid to settle. The model wings flapped back and forth around the root in the horizontal direction with constant velocity and at 150° amplitude. Stroke cycle time was 3.876 s. The timing of wing rotation at the stroke reversals was symmetrical each lasting ± 10% cycle time (Fig. 2b, c). The wing’s feathering angle was 45° during the up and down strokes and 0° (vertical position) at the stroke reversals. Mean Reynolds number based on wing length and wing tip velocity was ~ 3.4 in all experiments (Engels et al. 2021; O’Callaghan et al. 2022) and fluid temperature ~ 24 °C.

### 2-Dimensional particle image velocimetry (PIV)

To visualize vortices at the wing and in the wake, we seeded the glycerine by pumping air through a metal filter. The seeding consisted of small air bubbles with low mean upward velocity (~ 0.3 mm s^−1^) and high concentration. To minimize measurement errors associated with upward-drifting air bubbles, we waited approximately 1 h after seeding before starting flow visualization, allowing larger bubbles to disappear. The air bubbles were illuminated by a 50 mJ per pulse dual mini-Nd:YAG laser (Insight v. 5.1, TSI, USA) that created two identical positioned light sheets with ~ 3 mm thickness. Laser optics consisted of 1000 mm concave, –25 mm cylinder and –15 mm cylinder lenses. The timing between two laser pulses (∆t) varied depending on where the light sheet intersected with the wing to achieve 50% local overlap between the two PIV images, ranging from 9520 µs at the wing tip to 23,730 µs at the wing section nearest the root. The orientation of the light sheets was always perpendicular to the wing's longitudinal axis. For mapping the flow throughout the entire stroke cycle, we also used two self-built external delay circuitries that allowed us to trigger the laser pulses with various delays with respect to the onset of each flapping cycle.

The three-dimensionally printed PVA is not transparent. Wake and air bubbles on one side of the wing were thus shadowed, producing several missing fluid vectors during PIV analysis. Instead of combining PIV vectors of the two half strokes, we solved this problem using tilted mirrors (tilting angle 10–15°, Fig. 2a) behind the wing. These mirrors reflected the laser light that passed the wing above and below the stroke plane back into the PIV light sheet, allowing flow quantification of the entire fluid around the wing. Paired images of a 170 × 170 mm^2^ flow field were captured using a PowerView 2M camera equipped with a 50 mm lens (aperture 11, Nikkor AF, Nikon) and a green filter to reduce reflections from the ambient light. Data processing involved a two-frame cross-correlation of pixel intensity with a recursive Nyquist grid and a Hart correlator engine with bilinear peak engine. The interrogation area was 32 × 32 image pixels that resulted in 6,825 vectors per image. Vectors exceeding a tolerance of 6-times standard deviations were removed from the data set. We applied a neighbourhood mean filter with a tolerance of 2.0 ms^−1^ to the vectors and missing vectors were interpolated. At final post-processing, we applied a moderate smoothing algorithm with a neighbourhood size of 5 × 5 vectors and a Gaussian radius of 1.1.

To remove the flow component due to the wing's own flapping motion in the horizontal, we estimated the horizontal velocity for each wing section and subtracted this velocity component from all fluid vectors in the image. Some of the graphs in this study thus show the flow as the wing (animal) experiences the fluid and not the velocity that is seen by the observer outside the flow tank. As a consequence, the magnitude of velocity vectors near the wing is close to zero. Velocity subtraction and vorticity calculations were done by a TSI plug-in macro for Tecplot 9.0 (Tecplot, Inc., USA). To estimate mean fluid velocity, peak vorticity and circulation of vortical structures in a region-of-interest, we used a self-written software macro in Origin 7.0 (Microcal Origin, Inc, USA).

Although the flow during wing flapping is three-dimensional, fluid velocities in the spanwise direction of the wing (axial flow) were thought to be negligible because of low Reynolds number. This assumption is supported by detailed experimental and numerical analyses on the loss of enstrophy of the LEV in *Drosophila* model wings (see supplemental material in Lehmann et al. 2021). The latter study found that axial flow and vortex stretching are minimal during wing flapping, compared to other flow components. The study thus implied that two-dimensional measurements are sufficient to characterize the temporal development of vorticity. Remarkably, these data are valid for a Reynolds number 63-times higher than the one used in this study (~ 214 vs. ~ 3.4). At a Reynolds number of ~ 3.4, a 2-dimensional analysis of vorticity should thus be more than sufficient to estimate vortex strength during wing flapping. We averaged the measurements from the 3^rd^ to the 8^th^ stroke cycle (*N* = 6 cycles) because vorticity and forces are slightly higher in the first two stroke cycles after starting wing flapping from rest (O’Callaghan et al. 2022). If not stated otherwise, all presented data are means ± standard deviation.

## Results and discussion

### LEV development throughout the stroke cycle

**Fig. 3:**
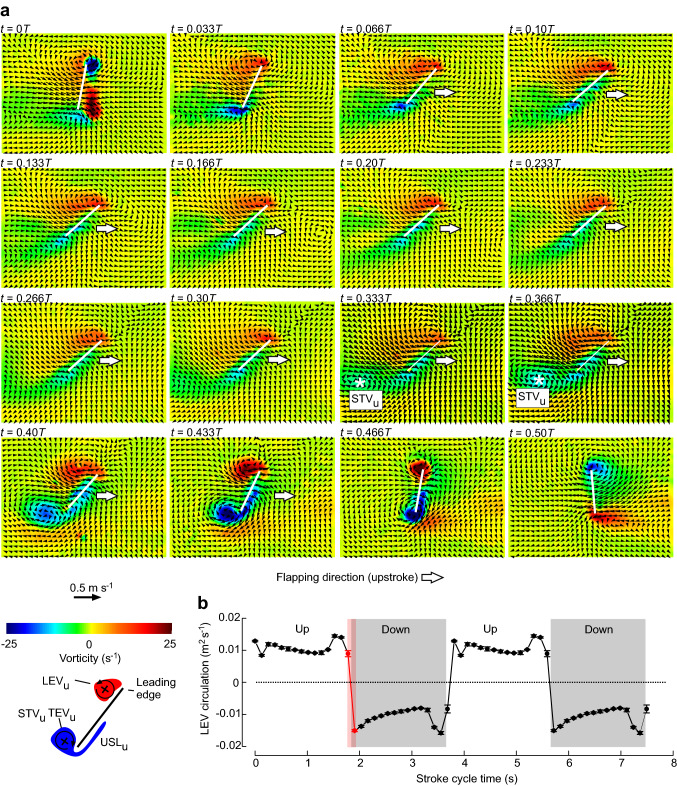
Temporal development of vorticity in bristled insect wings mapped throughout the stroke cycle (*T* = 0, 1). **a** Graph shows fluid flow during the wing’s upstroke and without subtraction of the free stream velocity (see materials and methods section). All tested wings developed a leading edge vortex (LEV), a trailing edge vortex (TEV) with a pronounced undershear layer (USL) and a start vortex (*, STV) during upstroke (u) and downstroke (d). White lines show the wing section at maximum wing chord (0.86 wing length). Due to low Reynolds number, the attached LEV is flattened and shed vortices such as the LEV at the end of each halfstroke and TEV at the beginning quickly dissipate. Schematics highlight vortex spin and position. **b** Temporal development of LEV circulation throughout the stroke cycle. Interrogation area of LEV vorticity was slightly adjusted to the location of each wing section. Data are duplicated for two cycles. Means ± standard deviation, *N* = 6 subsequent stroke cycles. Red indicates the time in which wing flapping direction reverses during the dorsal stroke reversal

All tested bristled wings produced a leading edge vortex and vertical downwash during wing flapping. Figure 3 shows vorticity and flow velocity at the wing and in the wake. As wing motion was identical during up- and downstroke, we only show the upstroke of the stroke cycle. The flow was captured at 30 equidistant points of time within the flapping cycle and, as expected from the low Reynolds number, flow was laminar without turbulence. This is similar to what has been described in computational fluid dynamic studies on the same wings and Reynolds number (Engels et al. 2020). All wings produced the leading edge vortex immediately at the beginning of each half stroke as the result of both, wing rotation and translation. Owing to low Reynolds number, the LEV is flattened and wraps around the wing leading edge. Figure 3 also shows a pronounced trailing edge vortex (TEV) and also vorticity in the undershear layer. Vorticity of the LEV is largest at the beginning of the half stroke and decreases approximately to 50% of its initial value during wing translation (Fig. 3b). There was no sign of a Wagner effect that describes the delayed development of steady-state circulation in a wing impulsively starting from rest at fixed angle of attack (Wagner 1925). Owing to the significance of viscosity, Fig. 3a also shows that the starting vortex from the upstroke (STV_u_) might not be fully shed from the trailing edge during translation as shown at *t* = 0.333* T* and *t* = 0.366* T*. This might suggest that bound circulation does not fully develop in each half stroke. An alternative explanation for the occurrence of STV_u_ is that it develops due to the wing's deceleration at the end of the downstroke. However, this explanation is not supported by the kinematics because horizontal flapping follows a sawtooth profile with constant velocity (Fig. 2b) and not a sinusoidal velocity profile. At the end of each half stroke, vorticity of LEV_u_ and TEV_u_ increase due to wing rotation but both vortices are not shed in the wake. This behaviour is different in hovering flight at higher Reynolds numbers and smaller viscous forces (e.g. Dickinson et al. 1999; Lehmann et al. 2021; Sun and Tang 2002; Sane 2003). The behaviour of LEV and TEV at the reversals are described in more detail in one of the following paragraphs.

### Significance of bristle spacing on LEV strength

**Fig. 4:**
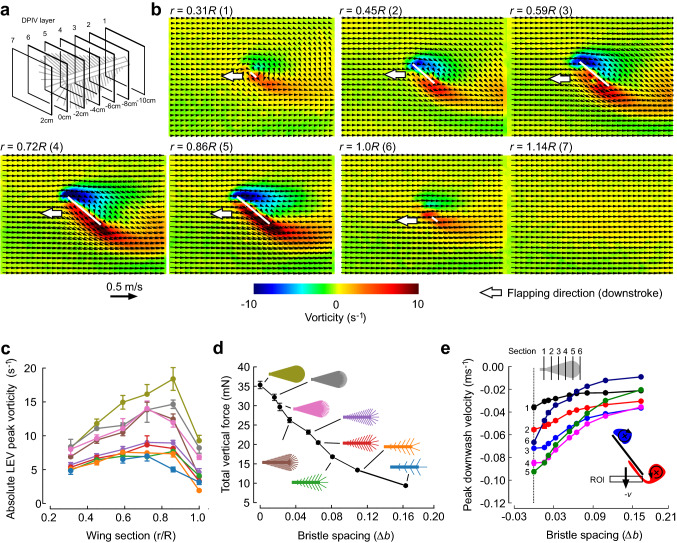
Vorticity and flow velocities during mid downstroke. **a** Location of the PIV slices corresponding to the distance *r* of total wing length *R* as shown in *b*. **b** Flow over the wings at 7 distances from the flapping axis. Local, horizontal free stream velocity (wing velocity) was subtracted from the flow vectors as described in the materials and methods section. Wing sections are shown as white lines. Numbers in parentheses correspond to the numbers in *e*. **c** Absolute LEV peak vorticity as a function of bristle spacing of the tested sections and all wings. Data are means from 6 consecutive stroke cycles (3rd–8th) ± standard deviation. **d** Lift-equivalent force production derived from LEV circulation (see main text). **e** Peak downwash velocity plotted as a function of bristle spacing. Vertical velocity (*v*) was estimated in a region-of-interest (ROI) below each wing section (see inset)

**Fig. 5:**
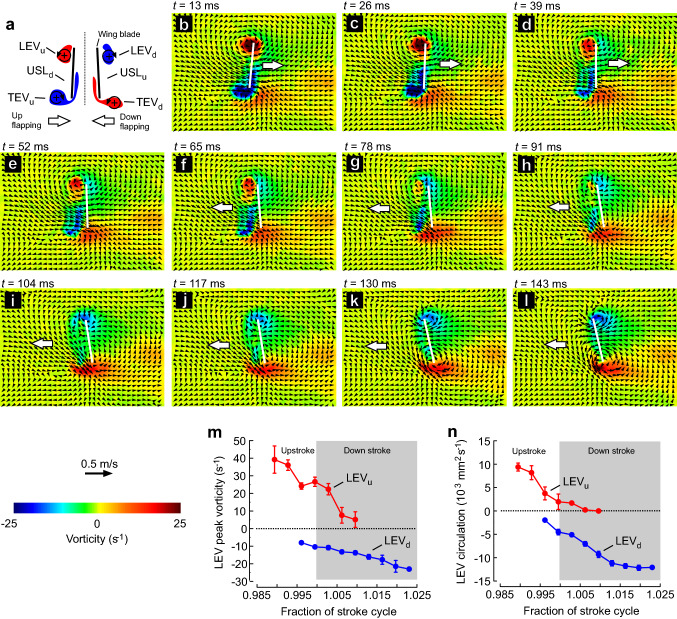
Time-resolved vorticity and flow velocities without subtraction of wing velocity during dorsal stroke reversal of wing motion. **a** Vortical structures. See legend to Fig. 3 for more explanations. **b–l** Behaviour of leading edge vortex, undershear layer and trailing edges vortex at the end of the upstroke and beginning of downstroke. **m** Peak vorticity of upstroke (downstroke) LEV ceases (develops) within approximately 2% of the stroke due to dissipation (wing rotation). **n** Temporal development of LEV circulation during wing rotation

To determine the decrease in aerodynamic performance of bristled wings, we scored LEV peak vorticity, LEV circulation and peak downwash velocity of all wings in Fig. 2d at mid down stroke and six wing sections (Fig. 4). One measured PIV plane captured the flow inside the tip vortex (*r * =  1.14*R*). Figures 4, 5 show flow patterns for a wing with bristle spacing ∆*b* = 0.016, because this wing is closest to the wing of *Gynaikothrips ficorum*. Figure 4c shows LEV peak vorticity for 6 wing sections and 9 tested wings. As expected from previous work, maximum vorticity of LEV and TEV occurs at maximum wing chord (*r * =  0.86*R*). The membranous wing performs best, reaching maximum vorticity of ~ 17.5 s^−1^ at 0.86*R*. The latter value converts into a wing chord and velocity normalized value of ~ 1700 m^−2^ (section velocity = 0.172 ms^−1^, wing chord = 0.06 m). With increasing bristle spacing, LEV peak vorticity decreases. To estimate overall performance of each wing from the LEV estimates of each wing section, we calculated force production from the product of LEV circulation, wing section velocity and fluid density, i.e.

1 $$L = \rho \sum\limits_{n = 1}^{6} {\left| {\Gamma_{LEV,n} } \right|} \left| {u_{\sec tion,n} } \right|\Delta r_{n}$$

with *L* denoting vertical force (lift), *ρ* the fluid density, *n* the number of wing section, *Γ* the circulation of LEV, *u* the translational velocity of the wing section, and ∆*r* the section width. Force production of each wing is plotted in Fig. 4d. The data show that lift production due to leading edge vorticity linearly decreases with increasing bristle spacing (linear regression fit on ∆*b* = 0–0.081, *N* = 7 wings, *R*^2^ = 0.99, *y* = − 0.223*x* + 0.035). As the inner wing shaft remains the same in all wings, the lift estimates saturate towards wings with large bristle spacing. Similar to the data in Fig. 4c, d, peak downwash velocity is highest in the membranous wing and at maximum wing chord (green, wing section 5, Fig. 4e). The drop in performance of the wing with Δ*b* = 0.016 (grey, Fig. 4d) compared to the fully membranous wing amounts to ~ 9%, while wetted wing area is only ~ 49% and wing mass ~ 26%, respectively. As inertial power requirements for flight (Ellington 1984) depend on wing mass, this data suggest that *Gynaikothrips* might save approximately half of the inertial power requirements for wing flapping at the cost of a 9% reduction in flight force production. A similar conclusion on the benefit of using bristled wings has been drawn from kinematic measurements and computational fluid dynamics analysis in the 395 μm large beetle *Paratuposa placentis* (Farisenkov et al. 2021).

### Behaviour of vortical structures

One of the major difficulties in force production by flapping wing motion is that vorticity produced in one half stroke must be reversed in the following (e.g. Dickinson et al. 1999; Sane 2003; Shyy et al. 2016, 2008). This means that LEV, TEV and bound circulation must be shed from the wing into the wake at the end of each half stroke. It has been shown that part of this circulation may be recycled by recapturing kinetic energy and enstrophy from the surrounding fluid (Dickinson et al. 1999; Lehmann et al. 2021). This even works in animals such as birds when flying in flocks, in which shed tip vortices from leading animals are re-used by animals flying behind (Weimerskirch et al. 2001; May 1979; Bajec and Heppner 2009). In water, trout save muscle power for swimming by recapturing vortices generated by bodies upstream (Liao et al. 2003). However, most of the energy is wasted at the stroke reversals and turned into heat by dissipation. The waste of energy is only one side of the equation, however, as vorticity must be shed quickly to allow the re-formation of vortices in the next half stroke.

To quantify the behaviour of LEVs at low Reynolds number in more detail, we mapped the flow during wing rotation with 13 ms resolution (~ 3.36 × 10^–3^ *t*/*T*, Fig. 5). Figure 5a–l shows that low Reynolds number impedes vortex shedding, keeping LEV and TEV close to the wing during the stroke reversals. This is different from wing rotation in larger insects such as the fruit fly (*Re* ~ 130, Lehmann et al. 2021) and wing models flapping at similar Reynolds number (Lehmann et al. 2005). A previously published study has shown that in fruit flies, the wing bends chordwise during the dorsal stroke reversal, trapping the upstroke LEV after it is shed during wing rotation. The trapped LEV subsequently travels along the ventral wing surface while the wing continues to rotate and transfers its enstrophy to the wing. Data show that this may improve flight efficiency in fruit flies (Lehmann et al. 2021). By contrast, in rigid fruit fly model wings, the upstroke LEV is also shed but remains close to the leading edge of the wing during the reversal (Lehmann et al. 2005). At Reynolds numbers of ~ 130–140, dissipation is comparatively small and vortices do not lose much of their enstrophy during the time of reversal. This has been shown by numerical and experimental work (supplemental information in Lehmann et al. 2021).

Figure 5m, n suggests that at low Reynolds number flight with a bristled wing (∆*b* = 0.016), the LEV from the upstroke dissipates surprisingly quickly within only ~ 2% of the stroke cycle time, while total time of wing rotation amounts to ± 10% (see materials and methods section). This elevated dissipation makes vortex shedding obsolete during the reversal and allows a quick build-up of counter-vorticity when the wing reverses flapping direction. As already mentioned above, it is less likely that the measured vorticity decay in a two-dimensional PIV slice is due to a shift of the conical LEV in the axial direction and CFD analysis has also shown that vortex stretching is negligible at Reynolds number below 140 (Lehmann et al. 2021). We thus fully attribute the measured LEV decay to the fluid’s viscous forces. Figure 5n shows that circulation of the following downstroke LEV (blue, Fig. 5h–l) reaches its maximum within ~ 2% stroke cycle time or ~ 78 ms of the 3.876 s stroke cycle, which is similar to the time it takes to dissipate the LEV from the upstroke.

## Conclusions

Many previous experimental studies focussing on insect flight investigated wing morphology and flapping aerodynamics in large insects with membranous wings, and at Reynolds numbers ranging from ~ 140 (e.g. fruit flies) to several thousands (e.g. dragonflies, moths, butterflies and grasshoppers). Bristled wings exclusively occur in tiny insects with only one known exception, wherefore local changes in flow conditions at the wing resemble fluid dynamic phenomena typical for low Reynolds numbers. Our data support previous findings that bristled insect wings are always less effective for aerodynamic lift production (Lee et al. 2018, 2020; Engels et al. 2021). Figure 4 shows that this is in part due to the decrease in LEV strength. Membranous and bristled wings co-exist in small insects, although bristled wings reduce inertial power requirements during wing flapping (Ellington 1984; Dudley 2000). The relative benefit of bristled wings for inertial costs, however, depends on the ratio between aerodynamic and inertial costs and thus on many other aspects of the flight motor. In conclusion, flight at low Reynolds number is costly and even small morphological adaptations in wing design which increase aerodynamic efficiency in flight should be beneficial for the evolution of miniaturized insects.

## Data Availability

All data of this study are available on request by contacting the corresponding author.
